# Does activation of the protective Renin-Angiotensin System have therapeutic potential in COVID-19?

**DOI:** 10.1186/s10020-020-00211-0

**Published:** 2020-08-17

**Authors:** Pawel Namsolleck, Gert N. Moll

**Affiliations:** 1 Lanthio Pharma, a MorphoSys AG company, Rozenburglaan 13B, 9727 DL Groningen, the Netherlands; 2 Department of Molecular Genetics, Groningen Biomolecular Sciences and Biotechnology Institute, University of Groningen, Nijenborgh 7, 9747 AG Groningen, the Netherlands

**Keywords:** COVID-19, ARDS, ACE2, Angiotensin, AT_1_R, AT_2_R, MasR

## Abstract

Infection of lung cells by the corona virus results in a loss of the balance between, on the one hand, angiotensin II-mediated stimulation of the angiotensin II type 1 receptor and, on the other hand, stimulation of the angiotensin II type 2 receptor and/or the Mas receptor. The unbalanced enhanced stimulation of the angiotensin II type 1 receptor causes inflammation, edema and contributes to the pathogenesis of severe acute respiratory distress syndrome. Here we hypothesize that stable, receptor-specific agonists of the angiotensin II type 2 receptor and of the Mas receptor are molecular medicines to treat COVID-19 patients. These agonists have therapeutic potential in the acute disease but in addition may reduce COVID-19-associated long-term pulmonary dysfunction and overall end-organ damage of this disease.

Recent publications highlight ACE2 as a cell-entry receptor for SARS-CoV and SARS-CoV-2. Less attention is given to other, in particular protective, components of the Renin Angiotensin System (RAS) (Unger et al. 2015). RAS has a double nature, like the two-faced ancient Roman god Janus, which simultaneously looks in opposite directions. The Detrimental Arm of RAS is formed by the ACE-Angiotensin II (Ang II)-angiotensin II type 1 receptor (AT_1_R) axis. Limiting the detrimental effects of AT_1_R by AT_1_R blockers (ARBs) or by inhibiting RAS via ACE inhibitors (ACEi) is generally well-established. However, the use of ARBs and ACEi in coronavirus disease-2019 (COVID-19) has been subject of debate. On the other hand, as part of the Protective Arm of RAS, Ang II also stimulates the angiotensin II type 2 receptor (AT_2_R) and this octapeptide can be further cleaved by the carboxypeptidase ACE2 to yield angiotensin-(1–7) (Ang-(1–7)), an agonist of the Mas receptor (MasR). The protective effects of AT_2_R and MasR agonists are usually opposite to the detrimental effects of AT_1_R, but their clinical use, in cases of unbalance between the two Arms of RAS, is insufficiently explored. Endogenous ligands of the RAS receptors are rapidly degraded and lack receptor specificity. Here we consider therapeutic perspectives of stable and specific AT_2_R and MasR agonists in COVID-19.

The balance between the Detrimental and Protective Arm of RAS is in several aspects seriously disturbed in COVID-19, thus causing a potentially lethal disease (Fig. 1). After the SARS-CoV cell-entry following ACE2-interaction, subsequent down-regulation of cell surface ACE2 is observed (Kuba et al. 2005). Since SARS-CoV-2 also targets ACE2, likewise downregulation of ACE2 is expected. Reduced membrane expression of ACE2 enhances the inflammatory response to the virus. COVID-19 infection furthermore causes an increase in the decapeptide Ang I and the octapeptide Ang II, whereas Ang-(1–7) levels decrease. Thereby detrimental Ang II-mediated stimulation of AT_1_R is enhanced whereas protective Ang-(1–7)-mediated stimulation of MasR is decreased. AT_1_R stimulation reduces alveolar cell survival. It also causes inflammation and an increase in vascular permeability (Huertas et al. 2020). As a result, edema is accumulating in the alveoli which hampers gas-exchange leading to lower oxygen levels. Taken together this adds to the severity of the acute respiratory distress.

**Fig. 1:**
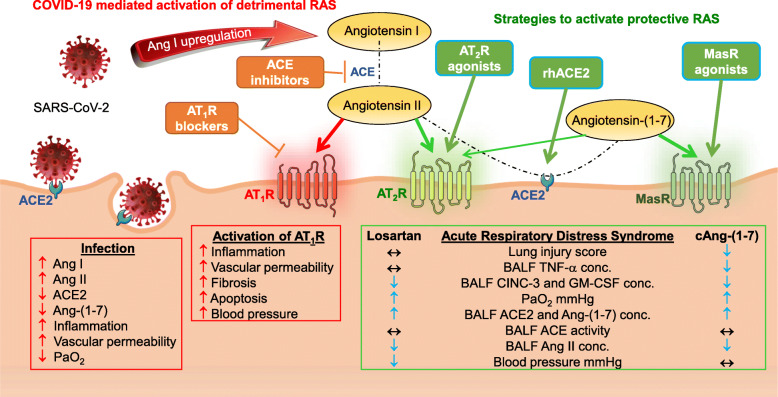
Potential treatments of SARS-CoV-2 infection within the Renin Angiotensin System containing a summary of an animal model of acute respiratory distress syndrome (Wösten-van Asperen et al. 2011)

Reduction of the unbalance in the RAS by inhibition of the Detrimental Arm might be reached by either an ARB or an ACEi. The combined use of ARBs and ACEi is prohibited, but their single use is applied. ARBs block the AT_1_R and thus Ang II can activate the unopposed protective receptor AT_2_R and further, after ACE2-mediated conversion of Ang II into Ang-(1–7), also the MasR. Unfortunately, ARBs exert only limited therapeutic effect in tissue injury (Unger et al. 2015). Moreover, ARBs may reduce blood pressure, which in case of critically ill patients may lead to unwanted hypotension. ACEi block the ACE-mediated cleavage of Ang I and thereby block the formation of Ang II. Pros and cons of the use of ARBs and ACEi in COVID-19 have been discussed (D'Ardes et al. 2020). Continuation of the use of an ARB or an ACEi in COVID-19 has been recommended (Vaduganathan et al. 2020; Ingraham et al. 2020; Park et al. 2020; Sanchis Gomar et al. 2020) and has been suggested to be beneficial in cardiovascular disease (Wang et al. 2020). Fear for induction of upregulation of the CoV-2-receptor ACE2 leading to enhanced infection (Sommerstein and Gråni 2020) has not been supported by clinical data (Gupta and Misra 2020; Kai and Kai 2020). In fact a clinical investigation demonstrated that no ARB or ACEi-induced upregulation of ACE2 takes place (Sriram and Insel 2020). On the other hand, benefits with respect to reducing COVID-19 itself have not (yet) been demonstrated in the clinic either (Gupta and Misra 2020; Kai and Kai 2020; Rico-Mesa et al. 2020). Instead of blocking AT_1_R or inhibiting ACE, here we focus on the potential benefits in COVID-19 of stimulating the AT_2_R or MasR.

Restoration of the balance in the RAS after corona virus infection might be pursued by direct and specific stimulation of the Protective Arm via AT_2_R or via the ACE2 - Ang-(1–7) - MasR axis. In a subchronic lung injury model a cyclized AT_2_R-specific peptide agonist, with a half-life of > 2 h in man, reduced inflammation and hypertrophy (Wagenaar et al. 2013). In an animal model of monocrotaline-induced pulmonary hypertension, a small molecule AT_2_R agonist C21 reversed pulmonary fibrosis and prevented right ventricular fibrosis. Furthermore C21 improved right heart function, decreased pulmonary vessel wall thickness, and reduced pro-inflammatory cytokines (Bruce et al. 2015). In a bleomycin-induced lung injury model prolonged administration of the AT_2_R agonist C21 prevented and attenuated pulmonary fibrosis, collagen deposition and lung remodeling. In addition C21 reduced inflammation, improved lung pressure and reduced muscularization of the pulmonary vessels (Rathinasabapathy et al. 2018). Currently the safety and efficacy of this agonist is tested in a Phase 2 trial with patients with COVID-19 infection (ClinicalTrials.gov Identifier: NCT04452435).

Recombinant human ACE2, which is not membrane bound, still binds to the corona virus and thereby limits the cell entry (Fig. 1). Furthermore recombinant ACE2 converts Ang II into Ang-(1–7). In patients with pulmonary arterial hypertension a single dose of recombinant human ACE2 resulted in a decreased level of pro-inflammatory cytokines and markers of oxidative stress accompanied by decreased pulmonary vascular resistance and increased cardiac output (Hemnes et al. 2018). To elucidate the molecular mechanisms leading to the observed effects, RNAseq on pulmonary arteries treated ex vivo with MasR agonist AVE0991 was performed. Significant changes in pressure regulation, inflammatory responses and cell migration pathways were observed indicating therapeutic effects of MasR activation (Hemnes et al. 2018). Stimulation of the MasR reduces in vitro Ang II- or bleomycin-induced apoptosis of alveolar epithelial cells (Uhal et al. 2011).

A recent review speculates on potential benefits of MasR stimulation in COVID-19 based on data obtained from animal models of asthma, lung fibrosis, ARDS, and pulmonary emphysema. The anti-inflammation effects, such as decreased cytokine and chemokine synthesis, migration of inflammatory cells to the lung and the resulting functional improvement of the lungs would be key benefits of MasR stimulation (Fig. 2). In addition, prolonged treatment might result in anti-fibrotic effects in lung tissue (Magalhaes et al. 2020).

**Fig. 2:**
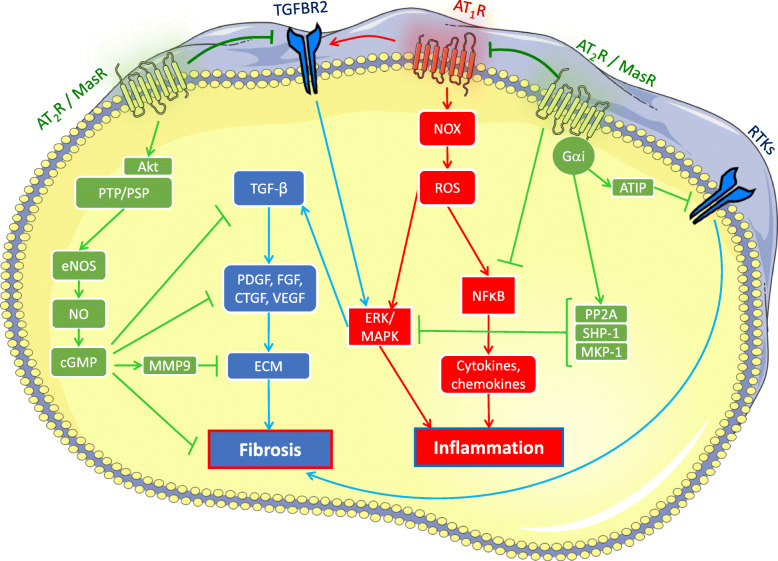
Anti-inflammatory and anti-fibrotic pathways mediated by activated AT_2_R and/or MasR. The AT_2_R and MasR are expressed in the cell as monomers, homodimers and AT_2_R-MasR heterodimers (Leonhardt et al. 2017) and their downstream pathways are largely similar, making it often impossible to distinguish between them. During infection the AT_1_R becomes activated initiating inflammatory processes via NFκB and MAPK. Prolonged activation of AT_1_R may initiate pro-fibrotic processes with TGFβ as a key molecule. Agonist-mediated stimulation of AT_2_R or MasR inhibits activation of NFκB and MAPK resulting in anti-inflammation. For the anti-fibrotic action the inhibition of receptor tyrosine kinase activity by dephosphorylation on the one hand, and activation of cGMP on the other hand, plays a crucial role. In addition, heterodimerization between AT_1_R and AT_2_R or MasR inhibits detrimental effects mediated by AT_1_R. Blue lines: pro-fibrotic pathways; red lines: pro-inflammatory pathways; green lines: anti-inflammatory or anti-fibrotic pathways. AT_2_R / MasR: angiotensin II type 2 receptor or Mas receptor or AT_2_R-MasR heterodimers; TGFBR2: transforming growth factor beta receptor II; AT_1_R: angiotensin II type 1 receptor; RTKs: receptor tyrosine kinases; Akt: protein kinase B; PTP: protein tyrosine phosphatase; PSP: protein serine/threonine phosphatase; eNOS: nitric oxide synthase 3; NO: nitric oxide; cGMP: cyclic guanosine monophosphate; MMP9: matrix metallopeptidase 9; TGFβ: transforming growth factor beta; PDGF: platelet-derived growth factor; FGF: fibroblast growth factor; CTGF: connective tissue growth factor; VEGF: vascular endothelial growth factor; ECM: extracellular matrix; ERK: extracellular signal-regulated kinases; MAPK: mitogen-activated protein kinase; NOX: NADPH oxidase; ROS: reactive oxygen species; NFκB: nuclear factor kappa B; Gαi: G protein alpha i subunit; ATIP: AT_2_R-interacting proteins/microtubule-associated scaffold proteins; PP2A: protein phosphatase 2A; SHP-1: Src homology region 2 domain-containing phosphatase-1; MKP-1: MAPK Phosphatase 1. The pathways are based on: Unger et al. 2015; Sumners et al. 2019; Zhang et al. 2014; Leonhardt et al. 2017; Chappell and Al Zayadneh 2017

The potential of the ACE2 - Ang-(1–7) - MasR axis has furthermore been recognized as witnessed by registered clinical trials of Ang-(1–7) in COVID-19 (ClinicalTrials.gov, Identifiers: NCT04332666; NCT04375124; NCT04401423). However, endogenous Ang-(1–7) lacks receptor specificity. Ang-(1–7) stimulates in vivo the MasR but in vitro studies reported biased agonism at the AT_1_R (Galandrin et al. 2016). In addition, Ang-(1–7) is very rapidly degraded resulting in a half-life of less than a minute in man. In contrast, specific and stable cyclic Ang-(1–7) exerts multiple therapeutic effects in lung tissue of animal models of acute and chronic lung injury (Wagenaar et al. 2013; Wösten-van Asperen et al. 2011).

In an animal model of ARDS, cyclic Ang-(1–7) reduced lung injury and inflammation while improving blood oxygenation (Fig. 1). Cyclic Ang-(1–7), which is fully ACE-resistant, did not change the blood pressure (Wösten-van Asperen et al. 2011). In addition to the acute and sub-chronic effects in COVID-19, stable AT_2_R agonists (Bruce et al. 2015) may reduce COVID-19-associated long term pulmonary dysfunction.

Besides the lungs, COVID-19 also affects heart, kidney, liver, gastrointestinal and the central nervous systems (Gan et al. 2020). In view of the demonstrated general therapeutic potential of the Protective Arm of RAS in these organs and systems (Unger et al. 2015), treatment of severe ARDS in COVID-19 with AT_2_R and MasR agonists may concomitantly confer beneficial effects that reduce the overall end-organ damage of this disease.

In conclusion, available data indicate the perspective of an effective strategy for treatment of ARDS and COVID-19 by direct and selective stimulation of the Protective Arm of RAS by AT_2_R- or MasR-specific, peptidase-resistant agonists. The data converge to further investigations in viral pneumonia-mediated ARDS models.

## Data Availability

Not applicable.

## Abbreviations

RAS: Renin angiotensin system
SARS: Severe acute respiratory syndrome
CoV-2: Coronavirus 2
COVID-19: Coronavirus disease 2019
ARDS: Acute respiratory distress syndrome
AT_2_R: Angiotensin II type 2 receptor
MasR: Mas receptor
ARB: Angiotensin II type 1 receptor blocker
ACE: Angiotensin converting enzyme
ACEi: Angiotensin converting enzyme inhibitor
Ang II: Angiotensin II
Ang-(1–7): Angiotensin-(1–7)
